# Clinical significance of nuclear non-phosphorylated beta-catenin in acute myeloid leukaemia and myelodysplastic syndrome

**DOI:** 10.1111/j.1365-2141.2007.06914.x

**Published:** 2008-02

**Authors:** Jinglan Xu, Momoko Suzuki, Yousuke Niwa, Junji Hiraga, Tetsuro Nagasaka, Masafumi Ito, Shigeo Nakamura, Akihiro Tomita, Akihiro Abe, Hitoshi Kiyoi, Tomohiro Kinoshita, Tomoki Naoe

**Affiliations:** 1 Department of Haematology and Oncology, Nagoya University Graduate School of Medicin Tsurumai-cho, Showa-ku, Nagoya, Japan; 2 Department of Clinical Pathophysiology, Nagoya University Graduate School of Medicine Tsurumai-cho, Showa-ku, Nagoya, Japan; 3 Department of Pathology, Japanese Red Cross Nagoya 1st Hospital Michishita-cho, Nakamura-ku, Nagoya, Japan; 4 Department of Infectious Diseases, Nagoya University Hospital Tsurumai-cho, Showa-ku, Nagoya, Japan

**Keywords:** beta-catenin, non-phosphorylated beta-catenin, acute myeloid leukaemia, myelodysplastic syndrome, immunohistochemistry

## Abstract

Wnt signaling activates the canonical pathway and induces the accumulation of non-phosphorylated beta-catenin (NPBC) in the nucleus. Although this pathway plays an important role in the maintenance of haematopoietic stem cells as well as in oncogenesis, the significance of nuclear NPBC remains unclear in malignant haematopoiesis. This study examined the expression of nuclear NPBC in bone marrow specimens from 54 and 44 patients with *de novo* acute myeloid leukaemia (AML) and myelodysplastic syndrome (MDS), respectively. On immunohistochemistry with an anti-NPBC antibody, the nuclei were positively stained in 22 and 18 of AML and MDS specimens, respectively. Staining of nuclear NPBC was associated with AML subtypes (M6 and M7), low complete remission (CR) rate, and poor prognosis. Nuclear NPBC was also associated with a high score when using the International Prognostic Scoring System (IPSS) for MDS and with −7/−7q and complex karyotypes. These findings suggest that *in situ* detection of nuclear NPBC by immunohistochemistry could provide new insights into the pathogenesis and prognosis of AML and MDS.

The Wnt/beta-catenin pathway is involved in the self-renewal and proliferation of haematopoietic stem cells (Reya *et al*, 2003; Willert *et al*, 2003). Signaling is initiated by binding of Wnt proteins to transmembrane receptors of the Frizzeled family (Giles *et al*, 2003). In the absence of Wnt signals, a dedicated complex of proteins that includes the tumor suppressor gene product APC, axin, and glycogen synthase kinase-3-beta (GSK3-beta) phosphorylates specific serine and threonine residues within the N-terminal region of beta-catenin, which leads to the ubiquitination of beta-catenin and its degradation by proteasomes (Conacci-Sorrell *et al*, 2002; Noort *et al*, 2002; Staal *et al*, 2002; Giles *et al*, 2003). Wnt signals block GSK3beta activity, resulting in the accumulation of non-phosphorylated beta-catenin (NPBC), which is finally translocated to the nucleus (Noort *et al*, 2002; Staal *et al*, 2002). Nuclear NPBC interacts with T-cell transcription factor (TCF) and lymphoid enhancer factor (LEF), and it activates target genes such as *MYC* and *CCND1* (He *et al*, 1998; Tetsu & Maccormick, 1999). Therefore, nuclear NPBC is known to be oncogenic in many solid tumors (Bienz & Clevers, 2000; Polakis, 2000). Mutations of APC, beta-catenin, or axin, which are observed in various tumors, lead to stabilization of NPBC (Morin *et al*, 1997; Barker & Clevers, 2000).

In the bone marrow (BM), Wnt proteins activate the beta-catenin pathway and the non-obese severe combined immunodeficient (NOD-SCID)-repopulating capacity of normal haematopoietic stem cells. They lead to increased expression of *HOXB4* and *NOTCH1* implicated in the self-renewal of haematopoietic stem cells (Reya *et al*, 2003). Up-regulation of the beta-catenin pathway has been suggested in chronic myeloid leukaemia (CML)-derived granulocyte-macrophage progenitor cells (GMPs) and multiple myeloma (MM) cells (Derksen *et al*, 2004; Jamieson *et al*, 2004). Furthermore, beta-catenin reportedly plays a significant role in promoting cell proliferation, adhesion, and survival *in vitro* (Chung *et al*, 2002). The expression of beta-catenin is also enhanced by oncogenic FLT3 signals and associated with poor prognosis (Tickenbrock *et al*, 2005; Ysebaert *et al*, 2006). However, there are some contradictory reports. Studies of conditional knock-out mice with a beta-catenin gene (*Ctnnb1*) deletion indicated that *Ctnnb1* is not indispensable for haematopoiesis (Cobas *et al*, 2004). Furthermore, an active form of *Ctnnb1* compromised haematopoietic stem cell maintenance and blocked differentiation in transgenic mice experiments (Simon *et al*, 2005). The role of the Wnt/beta-catenin pathway in malignant haematopoiesis therefore needs to be further elucidated.

According to previous studies, the expression of beta-catenin is associated with activation of the Wnt pathway as well as poor prognosis (Tickenbrock *et al*, 2005; Ysebaert *et al*, 2006). However, beta-catenin is associated not only with Wnt signaling but also with adherence junctions (Conacci-Sorrell *et al*, 2002). It is anchored to the cell inner surface membrane via cadherins. In normal bone marrow (BM), the vascular endothelium expresses a significantly higher amount of beta-catenin relative to the level in haematopoietic cells. Accordingly, immunohistochemical detection of nuclear NPBC would enable a better understanding of the role of beta-catenin in leukaemia.

This study investigated the subcellular localization of beta-catenin in BM specimens from acute myeloid leukaemia (AML) and myelodysplastic syndrome (MDS) patients using two anti-beta-catenin antibodies: one against C-terminal peptides and another against N-terminal non-phosphorylated peptides. The latter antibody detected nuclear NPBC, and positive staining for nuclear NPBC was associated with particular clinical characteristics of AML and MDS.

## Materials and methods

### Patient samples

For clinical samples, BM clots were obtained during routine diagnostic procedures. Beta-catenin expression was analyzed in BM specimens from patients newly diagnosed at the Nagoya University Hospital between 2000 and 2006 (Table I). The *de novo* AML patients consisted of 35 men and 19 women with a median age of 53 years (range, 20–81 years), and *FLT3* mutations were detected in seven of 22 patients with AML (31·8%). The MDS patients consisted of 28 men and 16 women with a median age of 57 years (range, 22–89 years). BM mononuclear cells were harvested by standard Ficoll/Paque density gradient centrifugation (Amersham Pharmacia Biosciences, Roozendaal, the Netherlands), and were suspended in RPMI 1640 medium supplemented with 10% fetal bovine serum, 100 IU/ml of penicillin G and 100 μg/ml of streptomycin.

**Table I:** Clinical characteristics of AML and MDS patients according to nuclear NPBC expression.

	Nuclear NPBC^+^	Nuclear NPBC^-^	*P*-value
Patients with*de novo* AML	22	32	
Age (median)	54 (20–81)	53 (18–71)	NS
Sex/male/female	18/4	17/15	0·005
Laboratory data			
WBC (×10^9^/l; median)	3·5 (0·8–92·5)	5·3 (0·7–202·1)	NS
Hb (g/l; median)	74 (43–134)	98 (36–141)	0·01
PLT (×10^9^/l; median)	7·6 (0·3–170)	4·2 (1·1–27·3)	NS
PB blasts (%; median)	24 (0–82)	39 (0–99)	NS
BM blasts (%; median)	46·2 (20–86·5)	77·5 (29–98)	0·02
CR rate	13/22 (59·1%)	24/27 (88·9%)	0·01
Relapse rate	16/21 (76·2%)	14/24 (58·3%)	0·03
Patients with MDS	18	26	
Age	59 (26–76)	57 (22–89)	
Sex/male/female	12/6	16/10	0·05
Laboratory data			
WBC (×10^9^/l; median)	2·9 (1·2–9·0)	2·6 (1·6–5·9)	NS
Hb (g/l; median)	75 (46–151)	85 (47–127)	NS
PLT (×10^9^/l; median)	79 (7–122)	44 (7–400)	NS
BM blasts (%; median)	3 (0–30)	5 (0–30)	NS
IPSS score*			
Low risk	0	4	NS
Intermediate-1	4	12	NS
Intermediate-2	4	4	NS
High risk	3	1	0·04

NPBC, non-phosphorylated beta-catenin; AML, acute myeloid leukaemia; MDS, myelodysplastic syndrome; WBC, white blood cell count; Hb, hemoglobin concentration; PLT, platelet count; PB, peripheral blood; BM, bone marrow; CR, complete remission; IPSS, International Prognostic Scoring System.

* Full data was available in 32 of the 44 patients with MDS.

### Antibodies

For immunohistochemical and immunoblot studies, two monoclonal antibodies were used; one was against C-terminal peptides (clone14, IgG1; BD Transduction Laboratories/Life Science Research, Heidelberg, Germany), enabling recognition of pan beta-catenin (PBC), and the other was against N-terminal-peptides (clone 8E4, IgG1; Alexis Biochemicals, Lausanne, Switzerland), enabling recognition of NPBC.

### Immunohistochemical staining

Samples were fixed with ice-cold 4% paraformaldehyde for 16–24 h, embedded in paraffin, sectioned transversely (thickness, 3 μm), and processed for immunohistochemistry to determine the localization of beta-catenin. After removal of paraffin with xylene and dehydration with a series of ethanol solutions, the tissue sections were subjected to microwave irradiation (750 W) for 15 min in 0·01 mol/l citrate buffer (pH 6·0). The sections were then placed in an automated immunostainer (Ventana Medical Systems, Tucson, AZ, USA) as described (Xu *et al*, 2002). For negative controls, primary antibodies were replaced with mouse IgG. The subcellular distribution of beta-catenin (i.e. restriction to the nucleus or presence in the membrane) was assessed without knowledge of the French-America-British (FAB) subtypes, *FLT3* mutations or karyotypes. We investigated a single case twice for NPBC expression. The entire section was screened to find the region with the highest immunostaining. The score was determined in each case after counting at least 500 nuclei in 3–5 randomly selected regions. When 20% or more of the BM mononuclear cells were positive for nuclear staining of NPBC, they were classified as nuclear NPBC^+^. The cut-off value of 20% was determined by the median distribution of the percentage of BM mononuclear cells stained by NPBC. As described in the results, some erythroblasts were positive for NPBC but the number was <20% except in the case of M6 patients. On the other hand, almost all blasts in M7 and other cases tested positive. The discrimination of cell types based on the 20% criterion therefore enabled a clear delineation.

### Immunoblotting

Cell lysates from AML cells were extracted as previously described (Ozeki *et al*, 2004). A total of 1 × 10^6^ cells were directly lysed in sample buffer and then subjected to sodium dodecyl sulphate-polyacrylamide gel electrophoresis on a 10% gel, and the separated proteins were transferred to a polyvinylidene difluoride membrane (Bio-Rad, Hercules, CA, USA). The membrane was initially incubated at room temperature for 1 h with 5% nonfat milk and 0·1% Tween-20 in Tris-buffered saline and then overnight with mouse monoclonal antibodies at a 1:2000 dilution in the same solution. After washing, the membrane was incubated for 1 h with a 1:5000 dilution of horseradish peroxidase-conjugated mouse antibodies to mouse IgG (MBL, Amersham, Bucks, UK), and immune complexes were then detected with enhanced chemiluminescence (ECL) reagents (Amersham).

### Statistical analysis

The χ^2^ test was used to calculate the difference of frequencies between nuclear NPBC^+^ and NPBC^−^ groups. The Mann–Whitney *U*-test was used to compare continuous variables. Kaplan–Meier curves were drawn using statview software (Macintosh; SAS Institute, Cary, NC, USA). *P-*values <0·05 were considered significant.

## Results

**Fig 1:**
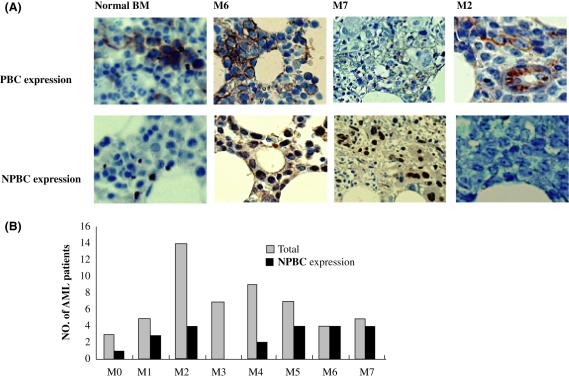
Specific association of NPBC expression with FAB subtypes of AML specimens. (A) In normal BM clots, PBC is expressed on the membranes of erythroid cells as well as endothelial cells. NPBC was not detected in normal BM clots. Most erythroid cells and endothelial cells showed cell membrane expression of PBC without expression in the nucleus or cytoplasm. NPBC was expressed in leukemia cells and was always restricted to the nucleus, especially in M6 and M7 specimens. Prominent staining of endothelial cells was seen in the vascular tissue in BM derived from an M2 patient with, though NPBC staining was negative in the same specimen. Original magnification ×40. (B) The graph presents data based on nuclear NPBC staining in paraffin sections from 54 patients with AML.

Using an anti-beta-catenin C-terminal peptide antibody, beta-catenin was stained in the membrane and cytoplasm of erythroid cells from normal BM. In *de novo* AML specimens, significant staining was observed only in M6. This antibody also detected BM vessels whose density was increased in AML specimens as previously reported (Serinsöz *et al*, 2004; Fig 1A). On the other hand, an anti-N-terminal nonphosphorylated peptide antibody gave no significant staining in the normal BM. In AML specimens, nuclear NPBC was detected in erythroid blasts, megakaryoblasts and some myeloblasts (Fig 1A). In M6 specimens, nuclear NPBC was detected in 30–80% of myelomonocytic cells and nearly 100% of erythroblastic cells (Fig 1A). In M7 specimens, megakaryocytes were also strongly positive for nuclear NPBC (Fig 1A). In total, 20% or more of the BM mononuclear cells were positive for nuclear NPBC in 22 (40·7%) of 54 AML patients (Table I, Fig 1B). There was a strong male predominance of nuclear NPBC^+^ cases, comprising 81·8% (18/22) in AML patients (Table I). However, the reason for this is unclear. In our cohort study, the karyotypes of female patients correlated to t(8;21)/t(15;17), which did not express nuclear NPBC. Thus the small numbers of studied patients seem to give some bias to the male predominance. A large-scale study is necessary to confirm this association. Nuclear NPBC^+^ staining was closely associated with AML subtype: it occurred frequently (8/9) in M6 and M7 and rarely (0/7) in M3 (Fig 1B), and nuclear NPBC^+^ was preferentially detected in erythroid and megakaryoblastic leukaemia compared to other myeloid leukaemias (M6–M7 vs. M0–M5, *P* < 0·001).

**Fig 2:**
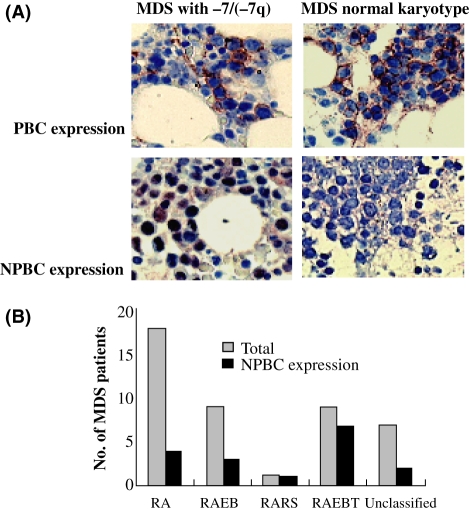
Specific association of NPBC expression with FAB subtypes of MDS specimens. (A) Most erythroid cells and endothelial cells showed cell membrane expression of PBC without expression in the nucleus or cytoplasm. NPBC was expressed in erythroid cells and was always restricted to the nucleus, especially in refractory anaemia with excess blasts in transformation (RAEBT) and MDS specimens with −7/−7(q). Original magnification ×40. (B) The graph presents data obtained for nuclear NPBC staining in paraffin sections from 44 patients with MDS. RA, refractory anaemia; RARS, RA with ringed sideroblasts; RAEB, RA with excess blasts.

In MDS specimens, erythroid cells and endothelial cells were stained with the anti-beta-catenin C-terminal peptide antibody (Fig 2A). As observed for AML specimens, the cytoplasm and inner membrane were stained by this antibody. The anti-beta-catenin N-terminal nonphosphorylated peptide antibody detected nuclear staining in myeloblasts and erythroblasts that was similar to the pattern seen in AML cases (Fig 2A). Nuclear NPBC was found in 18 (40·9%) of 44 MDS patients, and was related to the FAB classification of MDS (Table I, Fig 2B). Nuclear NPBC^+^ was preferentially detected in refractory anaemia with excess blasts in transformation (RAEBT) compared to other MDS subtypes [RAEBT *versus* refractory anaemia (RA)/refractory anaemia with ringed sideroblasts (RARS)/RAEB, *P* = 0·01].

**Fig 3:**
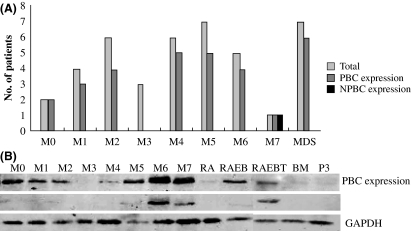
Beta-catenin expression of AML cells assessed by immunoblotting. (A) The graph shows data obtained for the expression of NPBC in mononuclear cells from 41 patients with AML, and the data indicate that expression of NPBC is specific to some FAB subtypes, especially to M6 and M7. (B) Representative immunoblots for PBC and NPBC in AML samples. RA, refractory anaemia; RARS, RA with ringed sideroblasts; RAEB, RA with excess blasts; RAEBT, refractory anaemia with excess blasts in transformation.

To confirm whether these two antibodies recognized beta-catenin, a total of 41 samples from AML and MDS patients were subjected to immunoblot analysis. The anti-beta-catenin C-terminal peptide antibody detected bands at a molecular weight of 95 kDa, corresponding to beta-catenin, in most samples except for M3 (Fig 3A). The anti-N-terminal nonphosphorylated peptide antibody gave bands of the same size in only a few samples of AML and MDS (Fig 3B). The results of immunoblotting corresponded to those of immunostaining, although the latter was more sensitive than the former.

The above findings suggest that expression of nuclear NPBC could be used to identify some subsets of AML and MDS. Next we studied whether nuclear NPBC was associated with chromosomal abnormalities or genetic alterations. Previous studies suggested that AML-associated translocations, such as t(8;21) and t(15;17), contributed to the activation of gamma-catenin, or that *FLT3* mutation might be associated with the stabilization of beta-catenin. In this study, however, nuclear NPBC was never detected in AML with t(8;21) or t(15;17). In AML/MDS with −7/−7q and a complex karyotype, nuclear NPBC was frequently detected (*P* = 0·007 and *P* = 0·02, respectively; Table II). Moreover, detection was not related to *FLT3* internal tandem duplication (ITD; Table II).

**Table II:** Cytogenetic abnormalities and *FLT3* mutation according to nuclear NPBC expression.

	Nuclear NPBC^+^ (*N*)	Nuclear NPBC^−^ (*N*)	*P*-value
Karyotypes			
t(8;21)	0	3	NS
t(15;17)	0	4	NS
−5/−5q	4	1	NS
−7/−7q	12	6	0·007
Complex	13	7	0·02
Others	19	2	NS
Normal	10	31	0·0003
Unknown	7	1	0·02
*FLT3* mutation			
Wild type	3	12	0·006
ITD	2	5	NS

Patients are counted more than once due to the coexistence of more than one cytogenetic abnormality. Complex: patients had three or more cytogenetic abnormalities.

NPBC, non-phosphorylated beta-catenin; ITD, internal tandem repeat; NS, not significant.

**Fig 4:**
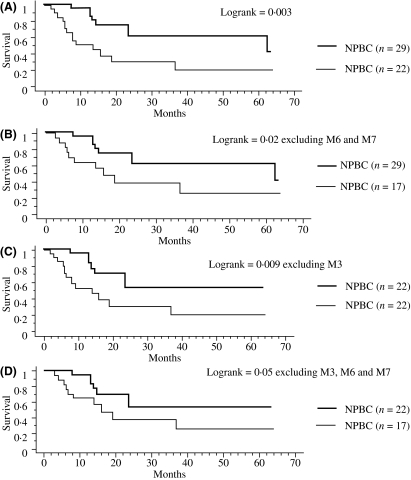
Kaplan–Meier cumulative survival curves were calculated for 51 AML patients (A) and 46 patients excluding subtype M6/M7 (B), 44 patients excluding subtype M3 (C), 39 patients excluding subtype M3/M6/M7 (D), respectively, according to the presence of nuclear NPBC. Comparison of the survival curves using the log-rank test identified nuclear NPBC^+^ as a prognostic factor.

Finally, we studied whether clinical characteristics and outcome were different between nuclear NPBC^+^ and NPBC^−^ AML patients. NPBC^+^ AML patients showed significantly lower hemoglobin levels, lower blast percentages in the BM, and lower CR rates (Table I). There were no significant differences between the NPBC^+^ and NPBC^−^ groups in the MDS patients (Table I). However, nuclear NPBC was associated with a high International Prognostic Scoring System (IPSS) score (Table I). Of note, nuclear NPBC^+^ AML patients had worse overall survival than NPBC^−^ AML patients (Fig 4A). Even if the M6/M7 subtype and/or M3 subtype was excluded from the analysis, there was still a significant association between nuclear NPBC^+^ and survival (Fig 4B–D).

## Discussion

This study used nuclear NPBC as a biomarker for the activated Wnt/beta-catenin signaling pathway. The anti-beta-catenin C-terminal peptide antibody detected total beta-catenin in immunoblots but mainly cytoplasmic and membrane-associated beta-catenin in immunohistological analysis, whereas the anti-beta-catenin N-terminal nonphospholylated peptide antibody detected only nuclear beta-catenin in both analyses (data not shown). Accordingly, it was concluded that the nuclear staining with the latter antibody identified nuclear non-phosphorylated beta-catenin. Nuclear NPBC was detected in 22 (40·7%) of 54 AML patients and 18 (40·9%) of 44 MDS patients. Positive staining of nuclear NPBC was associated with particular AML subtypes (M6 and M7), a low complete remission rate, and poor prognosis. The presence of nuclear NPBC was also associated with a high IPSS score of MDS and with −7/−7q and complex karyotypes.

Previous reports indicated that a significant proportion of AML samples expressed beta-catenin on immunoblot analysis. The expression of beta-catenin is related to CD34 expression, poor prognosis and clonogenic capacity *ex vivo* (Ysebaert *et al*, 2006). Furthermore, beta-catenin is expressed in normal CD34^+^ progenitor cells and the expression level is reduced upon differentiation (Simon *et al*, 2005). In these studies, however, the total beta-catenin level was analyzed only by immunoblot analysis. Beta-catenin is expressed not only as nuclear NPBC but also as a cadherin-associated protein in the inner cytomembrane (Conacci-Sorrell *et al*, 2002). The present study found that normal erythroblasts expressed cytoplasmic or membrane-associated beta-catenin but not nuclear NPBC (Fig 1A). Both membrane-associated and nuclear beta-catenin was expressed in malignant erythroblasts in subtype M6 (Fig 1A). These findings suggest that increased nuclear NPBC levels were the result of aberrant signal transduction in the Wnt pathway or an abnormality of beta-catenin itself.

In this study, the expression levels of total and non-phosphorylated beta-catenin were correlated but varied significantly among leukemia samples. In M6 and M7 samples, the expression of beta-catenin was significantly augmented, whereas it was hardly detected in M3 and normal BM samples. These variations suggest the possibility that Wnt/beta-catenin signaling is mediated by multiple factors, such as immaturity, lineage, and oncogenic signals.

We established a correlation between nuclear NPBC^+^ and poor survival in AML patients. The prognostic value of total beta-catenin expression has been previously studied in AML patients (Ysebaert *et al*, 2006), but the present study clearly showed for the first time that nuclear NPBC is associated with prognosis. The association was still observed even if M6/M7 and/or M3 patients were excluded from the analysis. Nuclear NPBC might be a new prognostic marker for AML and MDS that can be evaluated by histopathological examination.

Wnts are a family of paracrine and autocrine factors that regulate cell growth and cell fate (Reya *et al*, 2003). The Wnt autocrine signaling mechanism was initially discovered in human breast and ovarian tumor cell lines as well as in MM primary samples (Bafico *et al*, 2004; Derksen *et al*, 2004). Since several Wnt family members have been reported in BM stromal cells, it is possible that leukaemia cells respond to different proteins of the Wnt/beta-catenin pathway secreted by stromal cells in a paracrine fashion (Austin *et al*, 1997; Van Den Berg *et al*, 1998; Etheridge *et al*, 2004). Nuclear NPBC was detected in some cell lines only when they were transplanted in non-obese diabetic/severe combined immunodeficient/gammacnull (NOG) mice (data not shown). Thus, the leukaemia niche may have an important role in nuclear NPBC expression during AML.

MDS is a clonal hematopoietic stem cell disorder characterized by multi-lineage dysplasia and pancytopenia in which further genetic events may be required for the rapid expansion of leukaemic blasts (Heaney & Golde, 1999; Hirai, 2003). Although nuclear NPBC has been studied in many cancers as well as haematological malignancies, it has not been studied in MDS (Morin *et al*, 1997; Barker & Clevers, 2000; Giles *et al*, 2003). This is the first study to report the expression of beta-catenin in MDS patients. Here we showed that nuclear NPBC was related to the IPSS score and that secondary AML from MDS showed the highest percentage of nuclear NPBC expression. The Wnt signaling pathway may play an important role in the pathogenesis of the transformation of MDS into AML. Regarding chromosomal abnormalities, −7/−7q and/or complex karyotypes were significantly associated with the presence of nuclear NPBC. According to recent data (Liu *et al*, 2006), the gene encoding alpha-catenin (*CTNNA1*) is suppressed by deletion and/or methylation. Both alpha-catenin and beta-catenin bind to the inner membrane of hematopoietic cells, and cadherin binds to actin filaments via the catenin complex. If the expression of alpha-catenin is suppressed in the 5q- genotype or for other reasons, beta-catenin may be abnormally located or activated. In this study, abnormalities of chromosome 5 were associated with the presence of NPBC but this was not statistically significant. The reason why NPBC is associated with chromosome 7 abnormalities remains unclear. A gene encoded by chromosome 7 may be associated with the regulation of the Wnt/beta-catenin pathway. Several possible candidate genes including *SFRP4*, *WNT2*, and *FZD1* and *FZD9* are located at human chromosome 7. There are reports that sFRP4 plays a role in tumor suppression via the Wnt pathway (Hrzenjak *et al*, 2004; Horvath *et al*, 2007), although the specific relationship remains unknown. Since many molecules are directly or indirectly associated with the phosphorylation, stabilization and nuclear translocation of beta-catenin, the presence of nuclear NPBC might provide a clue to find a new leukemia-associated signaling mechanism.

In conclusion, *in situ* detection of nuclear NPBC by immunohistochemistry of paraffin sections from BM specimens could be used to predict the prognosis of AML and MDS. Understanding the mechanisms leading to leukemogenesis in nuclear NPBC^+^ AML and MDS may lead to new anti-leukemia therapies.
